# Rectal atresia: a rare cause of failure to pass meconium

**DOI:** 10.11604/pamj.2014.19.198.4057

**Published:** 2014-10-24

**Authors:** Fatima Zahrae Laamrani, Rachida Dafiri

**Affiliations:** 1 Radiology Department, Children's Hospital, Rabat, Morocco

**Keywords:** Rectal-atresia, failure to pass meconium, anorectal malformation

## Abstract

Rectal atresia or stenosis is an extremely rare anorectal malformation associating a normal anal canal with a stricture or a complete rectal atresia. We describe a case of rectal atresia in a newborn female presenting with an abdominal distension and failure of passing meconium.

## Introduction

Rectal atresia is a rare anorectal malformationcombining a normally developed anus and an atretic rectal segment. A failure to pass meconium in a new born with normal present anus must lead to further clinical and radiologic investigations which findings are specific (Table 1).

**Table 1:** Summary table of rectal atresia

Etiology	An in utero ischemic accident seems to explain the pathogenesis of this rectal malformation.
Incidence	1-2% of all anorectal anomalies
Gender Ratio	It occurs in both sex
Age predilection	Rectal atresia is revealed in the neonatal period
Treatment	There is an extensive list of creative operative procedures used for the rectal atresia reflecting the great difficulty faced in treating this anomaly
Prognosis	Continence is usually normal after reconstruction. Chronic constipation represents a common postoperative feature.
Imaging appearence	Barium enema shows the rectum with its proximal blinded pouch and the atretic segment which may interest any part of the rectum. Perineal ultrasonography and magnetic resonance imaging can be used to check preoperatively the exact relation between the anal canal and sphincters

## Patient and observation

**Figure 1:**
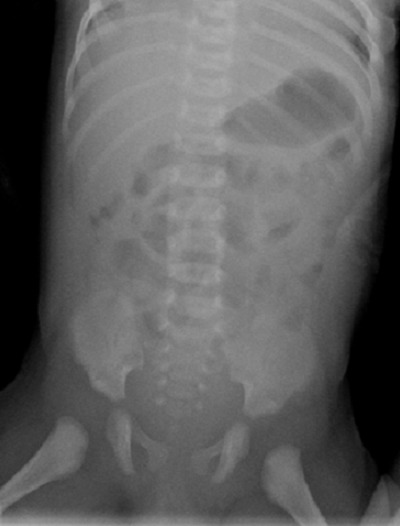
A twenty-day-old female child with a failure to pass to meconium. Technique and findings: Abdominal x–rays shows a massively distended colon with pelvic emptiness

**Figure 2:**
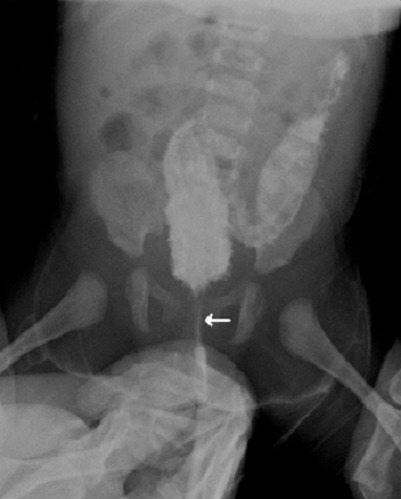
A twenty-day-old female child with a failure to pass to meconium. Technique and findings: Image from a barium enema study shows an atretic inferior rectal segment (arrow) with a superior rectal pouch with no fistula

A twenty-day-old female child was admitted with a history of chronic vomiting, not passing meconium since birth and a progressive abdominal distension. The perineal examination objectified a normally placed anus with no perineal fistula. When introducing a thermometerper rectum, it stops at 2 cm from the anal verge with no explosive stools. Abdominal x–raysshowed a massively distended colon with pelvic emptiness (Figure 1). A barium enema was performed, showing an atretic inferior rectal segment with a superior rectal pouch and no fistula (Figure 2). An end to end anastomosis after a posterior sagittal anorectoplasty was performed with a satisfying evolution.

## Discussion

### Etiology and demographics

Rectal atresia is a rare anorectal anomaly combining a normally developed anus and an atretic rectal segment representing 1-2% of all anorectal anomalies. Rectal atresia is considered separate from imperforate anus or anal atresia because, in rectal atresia, the anus is present and normal, but a variable rectal segment is atretic. Incomplete rectal atresia refers to complete membrane or severe stenosis. An in utero ischemic accident seems to explain the pathogenesis of this rectal malformation [1, 2].

### Clinical and imaging findings

Failure to pass meconium, progressive abdominal distention, refusal to feed and vomiting suggest the intestinal obstruction in neonates and lead to further investigations [3]. The clinical diagnosis is easy to confirm. When passing per rectum a firm catheter of size 8 or 10 stops at 2-3 cm from the anal verge [4]. After a rectal atresia is clinically identified, radiography must be performed and usually shows a colonic obstruction. Barium enema shows the rectum with its proximal blinded pouch and the atretic segment which may interest any part of the rectum [5]. Perineal ultrasonography and magnetic resonance imaging can be used to check preoperatively the exact relation between the anal canal and sphincters. They provide identification of both rectal pouch and sphincteric muscles without ionizing radiation risks [6].

### Treatment and prognosis

The extensive list of creative operative procedures used for the rectal atresia reflects the great difficulty faced in treating this anomaly. Optimal continence remains the crucial goal in the treatment of all forms of anorectal anomalies including rectal atresia in which the anal canal and sphincter are normally formed. Continence should be normal after reconstruction. Chronic constipation represents a common postoperative feature [1].

### Differential diagnoses (Table 2)

Differential diagnoses include all conditions associated to a low intestinal obstruction manifesting as a neonatalfailure to pass meconium: Hirschsprung's disease, meconium ileus, colonic atresia, small left colon syndrome, megacystis-microcolon-intestinal hyperperistaltism syndrome, anal atresia and all other anorectal malformations. Clinical and radiologic findings are specific and lead to positive diagnosis of rectal atresia [3].

**Table 2:** Table of differential diagnoses of rectal atresia

	Abdominal radiographs	Contrast Enema
Meconium ileus	Low intestinal obstruction that is characterized by multiple bowel loop dilatations with a relative lack of air-fluid levels within the dilated bowel loops because of the abnormally thick intra-luminal meconium.	Unused colon with multiple small filling defects representing meconium concretions.
Hirschsprung's disease	Low intestinal obstruction with multiple bowel loops.	May be completely normal or show an abnormal recto sigmoid ratio (<1), transition zone of rectal narrowing, irregular rectal contractions, and retained contrast materiel on delayed radiographs.
Colonic atresia	Multiple dilated bowel loops, multiple air-fluid levels, and absence of air in the rectum.	Distal unused colon with the more proximal dilated colon ending in a blind pouch.
Anal atresia (imperforate anus =the anus is absent or severely stenotic)	Low intestinal obstruction with multiple bowel loops. It also may be useful to determin whether the infant has a high or low anal atresia. (An ultrasonography can be performed to measure the distance between the perineum and the rectal pouch)	
Small left colon syndrome	Low intestinal obstruction with multiple bowel loops with air-fluid levels	-Shortened colon with a lack of the usual tortuosity from the anus to the splenic flexure/

## Conclusion

Rectal atresia is a rare anorectal anomaly combining a normally developed anus and an atretic rectal segment. Failure to pass meconium suggest the intestinal obstruction in neonates. Barium enema shows the rectum with its proximal blinded pouch and the atretic segment.
